# Identifying Video Game Preferences Among Adults Interested in Quitting Smoking Cigarettes: Survey Study

**DOI:** 10.2196/30949

**Published:** 2022-03-24

**Authors:** Caitlyn R Upton, Jessica A Nastasi, Bethany R Raiff

**Affiliations:** 1 Department of Psychology College of Science and Mathematics Rowan University Glassboro, NJ United States; 2 Department of Psychology College of Liberal Arts and Science University of Florida Gainesville, FL United States

**Keywords:** genres, popular games, smoking cessation, video games, smartphone, mobile phone

## Abstract

**Background:**

Smoking is the most prevalent cause of morbidity and mortality in the United States. Although most individuals who smoke express a desire to quit smoking, only a small percentage are successful. Serious games have become popular in health sectors as a potential avenue for delivering a scalable treatment that is both accessible and engaging for the smoking population. Several smoking cessation games have already been developed, but these games feature a broad range of gameplay elements and are not necessarily based on existing video game preferences in the general or smoking population.

**Objective:**

To better inform treatment development, this study aims to evaluate video game genre preferences among treatment-seeking individuals who smoke (N=473).

**Methods:**

Participants responded to a screening survey to enroll in a larger, serious game intervention for smoking cessation. During this screening survey, participants were asked to disclose their favorite video games, which resulted in 277 unique game titles. These titles were coded for genre categories based on publisher listings and game features. The genres were then analyzed for the frequency of reporting overall and across age groups.

**Results:**

Action, Role-Playing, and Action-Adventure were the most reported genres among adults aged ≤34 years; Action, Action-Adventure, and Logic were the most reported genres among adults aged 35-44 years; and Logic and Action were the most reported genres among adults aged ≥45 years.

**Conclusions:**

These data indicate that treatment-seeking individuals who smoke have different game preferences across age groups, and the data provide novel information to inform the development of future serious games targeting the smoking population that are tailored to the preferences of their age group.

**Trial Registration:**

ClinicalTrials.gov NCT03929003; https://clinicaltrials.gov/ct2/show/NCT03929003

## Introduction

### Background

More than 16 million adults in the United States are living with diseases related to cigarette smoking, and more than 480,000 deaths each year can be attributed to smoking-related causes [1]. The cost of direct medical care for conditions related to cigarette smoking is US $170 billion [2]. In 2018, more than half of adults in the United States (55.1%) who reported smoking had attempted to quit, but only 7.5% were successful [1,2], and quit rates remain low overall [3]. Given the substantial societal impact of cigarette smoking and the low success rates of those interested in quitting, there is a significant public health need to develop accessible and engaging methods of delivering smoking cessation interventions.

Serious games, or video games developed for purposes other than pure entertainment, have become popular in health sectors and may be one avenue for delivering a scalable treatment that is both accessible and engaging [4,5]. According to the Entertainment Software Association, 75% of households in the United States own a video game device, and 64% of adults play video games [6]. Several games have already been developed, targeting smoking cessation, such as *Cigbreak* [7], *Inspired* [8], *Tobstopp* [9], *QuitIt* [10], and *Quittr* [11], each of which uses different gameplay elements to facilitate smoking cessation. A recent review of 14 unique smoking prevention and cessation games not listed above found that of 7 smoking cessation game studies published, 5 had statistically significant positive results on smoking cessation outcomes—an indicator that serious games have a merit in smoking cessation research [12]. The review sought to identify common gameplay elements included in these games and found that the most frequently reported elements were rewards and punishment (eg, earning points for success and losing points for failure; n=32) and narrative and identity features (eg, the game follows a plot or story and the player uses an avatar to be integrated into the story; n=20) [12], but gameplay elements were inconsistently reported. In addition, of the published studies reviewed, the reasons participants may have dropped out or been unsuccessful were not reported, and it is possible that game genre or specific gameplay elements were an important factor. Derksen et al [12] suggested that the inconsistent reporting of gameplay elements makes it difficult to draw conclusions about each element and their combined impact on smoking cessation outcomes.

Publication bias may also limit the conclusions that can be made regarding the games that have already been developed but that were unsuccessful. There may also be more games in development or already commercially available that have not been studied empirically. As of 2017, there were 158 unique smoking cessation apps in the Apple App and Google Play stores; upon reviewing the 50 most popular apps, only 3 were supported by research [13]. There is also an array of mobile smoking cessation apps that use gamified elements (eg, diaries that provide feedback and rewards to users or games that seek to educate and distract individuals who smoke) that are available on the Google Play and Apple App stores but that have not undergone rigorous empirical testing. Thus, the number of evidence-based apps that are available to and approved by consumers and the ratio of evidence-based apps to nonevidence-based apps is lacking.

### Objectives

It is useful to know that serious games can create positive outcomes for individuals interested in quitting smoking, but this knowledge alone is not as useful as understanding specific components of games that may contribute to their success. Cugelman [14] outlined 7 criteria for game suitability for specific populations. Among the criteria were (1) identifying who the users of the game will be, (2) understanding the context of those users, (3) determining the compatibility of the intervention with the users, (4) identifying the goals of the intervention, and (5) deciding on the behavioral strategies that will be used. Part of the *context* noted above includes identifying the types of games that users are already familiar with and enjoy playing. For example, a smoker who plays *Fruit Ninja* might be more responsive to *Cigbreak* over *QuitIt* because of *Cigbreak*’s use of action mechanics that are similar to *Fruit Ninja* compared with *QuitIt*’s reliance on role-play. In this manner, game genre preferences (such as Action and Role-Play) can provide important information regarding the types of gameplay features that might be suitable for specific populations.

There are a substantial number of studies on genre preferences comparing across population characteristics such as age, gender, personality traits, and symptoms of behavioral disorders [15-21]. However, if one is seeking basic data about genre preferences in the general population, the sources of this information are derived from market research and nonpeer-reviewed sources [22,23]. For researchers seeking to develop games specifically for smoking cessation, there are currently no data to verify whether general population preferences match the preferences of treatment-seeking individuals who smoke. This study aims to identify the genres of video games that are most widely endorsed by a general population of treatment-seeking adults who smoked. By doing so, this study aims to provide a basis for future detailed examination of the gameplay elements and features that are frequently used in these genres. In the *Discussion* section, we have provided examples of basic gameplay features of the most popular genres and how those features might be leveraged for smoking cessation games.

## Methods

### Recruitment and Participants

Data collection was approved by the institutional review board (ProG0520140170), which oversees the protection of human subjects in research, as part of a larger research study that involved developing and testing a video game–based smoking cessation intervention. The data presented here were collected as part of a screening for that larger study, and because personally identifiable information was not collected at that stage, a waiver of consent was approved by the institutional review board. Participants were recruited via advertisements on Craigslist, Facebook, and Google Ads. A variety of advertisements were used, each with slightly different text and images to appeal to different audiences who might be interested in quitting smoking (eg, some advertisements showed images of a video game along with text about quitting, some showed images of parents with their children to appeal to people who might want to quit for loved ones, and others showed pictures of money to appeal to people who might want to quit for financial reasons). Those interested in participating followed the link to a screening survey to report information about smoking history, age, prior experience with video games, desire to quit smoking, access to mobile devices, and contact information.

Participants (N=473) were asked to list their favorite video games. Games listed had to meet the definition of a game as specified by Tekinbaş and Zimmerman [24]—a system in which players engage in an artificial conflict, defined by rules, that results in a quantifiable outcome—and list the specific name of a game or franchise of games. Out of the raw number of responses (N=473), there were a total of 337 game-related responses. Following data cleaning, 60 entries were excluded from the final analyses due to being broad genres rather than specific games (eg, *fighting games* and *soccer*) or not meeting the game definition (eg, a coloring book app called *Chamy*). A total of 277 unique games were included in this analysis.

### Procedure

Participants who answered the survey asked the open-ended question, “What is/are your favorite videogame(s)?” to determine prior experience with video games as well as questions about their smoking patterns (ie, number of cigarettes smoked per day and years spent smoking) and their age. Owing to the open-ended nature of the video game question, participants could list multiple games, allowing a greater representation of interests.

Each game was classified into genres identified by Adams [25]. The genres included Action, Action-Adventure, Adventure, Role-Playing, Simulation, Strategy, Sports, Massive Multiplayer Online (MMO), Party, Programming, Logic, Mobile, Trivia, and Board Games [25]. An additional category, Casino Games, was added because several participants reported playing casino and gambling games. Textbox 1 provides definitions of each category.

In total, 2 coders identified the genre of each individual game reported. Both coders searched for game titles using Google and, when available, used the genre reported by the publisher of the game. In the absence of publisher-identified genres, coders reviewed gameplay elements and store categorization to classify the game. The interobserver agreement was initially 77%. An additional coder who was not exposed to the previous coded list reconciled the 76 games that were scored differently between the primary coders. Reconciliation followed the same process described for the primary coders. The genre indicated by at least 2 of the 3 coders was selected as the official genre for the game and increased interobserver agreement to 99%. Participants’ ages were paired with their genre preferences to analyze trends across age groups using descriptive statistics.

Age bins were selected to match the Centers for Disease Control and Prevention National Adult Tobacco Survey age categories (18-24, 25-34, 35-44, 45-54, 55-64, and ≥65 years [1,2]). Crosstabs analyses and a 2-tailed Fisher exact test were performed to determine whether there were trends in game genres by age, with *α* set to *P*<.01. Fisher exact tests were performed comparing smoking intensity with genre preference. Participants were classified by the number of self-reported cigarettes smoked per day as light (<1 to 5 cigarettes per day), moderate (6-10 cigarettes per day), or heavy (>11 cigarettes per day) [26,27].

**Video game genres and definitions (definitions are from a book by Adams [25]).**

- **Action**: “Include physical challenges and require skills in hand-eye coordination to complete objectives, which include defeating opponents. (eg, *Call of Duty*)”
- **Action-Adventure**: “Involve components of both action (physical challenges, hand-eye coordination, opponent-based game play) and adventure elements (exploration, puzzle solving, relaxed time-constraints; eg, *Legend of Zelda*).”
- **Adventure**: “Focus on exploration of a story and environment through puzzles and interaction with other characters and the environment. There are no action challenges that require reflexes and these games do not demand the player use tactics and strategy to defeat an opponent. (eg, *Heavy Rain*)”
- **Role-Playing**: “Focus on growth of the player’s character through challenges and story, wherein the players character begins weak and steadily gains more experience, strength and access to better weapons as the game progresses. There is typically a hierarchy of opponents to defeat as part of a quest. (eg, *Final Fantasy*)”
- **Simulation**: “Involve players building things (such as in management and construction games) and/or playing through a simulated experience of life in the environments they build. They do not typically involve exploration, conflict, or physical challenges. (eg, *The Sims*)”
- **Strategy**: “Require skillful thinking, strategy and tactics to achieve goals such as building dynasty’s in a fantasy or real-world setting, or protecting a fortress from an invading force. (eg, *Civilization*)”
- **Sports**: “Simulate sports with players controlling a team or specific player, while another player or an artificial intelligence controls the opponents. (eg, *FIFA* series)”
- **Massive Multiplayer Online**: “An online multiplayer game that involves large numbers of online players in a virtual world. They can incorporate features of many different genres, and can be treated as a single or multiplayer experience. (eg, *World of Warcraft*)”
- **Casual, Mobile, Idle**: “Designed for short periods of play that can easily be entered and existed as needed. These can encompass a range of genre elements. Mobile refers to a game designed to be portable in nature (i.e., on a mobile phone). Idle games specifically feature a trivial task that a player accrues points over time for engaging in. (eg, *Angry Birds*)”
- **Party**: “Designed to support many players in a competition, but not on the same scale as an MMO. They include features such as racing and competition in small-groups. (eg, *Mario Party*)”
- **Logic**: “Require players to solve puzzles, navigate mazes, or match game images/tiles. Typically suited for casual play. (eg, *Candy Crush*)”
- **Casino**: “Prominently feature gambling elements such as slots and betting games, where the objective is to earn high points through risk. (eg, *Slots*)”
- **Board games**: “Classic board games such as chess, monopoly, or checkers that for which computerized version have been created.”
- **Trivia**: “Focus on answering questions to obtain points. (eg, *Words with Friends*)”

## Results

### Genres and Titles Reported

Participants were aged an average of 39 (SD 12.4; range 14-72) years, spent an average of 19.8 years smoking (SD 13.3; range 0-55 years), and smoked an average of 16.5 (SD 11.3; range 0-100) cigarettes per day. Table 1 shows the frequency with which different game genres were endorsed. Participants most often reported playing Action games (162/473, 34.2%), with titles such as *Call of Duty* and *Mario* being the most frequently endorsed. This was followed by Role-Playing (113/473, 23.9%), Action-Adventure (101/473, 21.4%), and Logic (79/473, 16.7%). Games identified as Action (57/277, 20.6%) had the most frequent variety of titles reported, followed by Role-Playing (51/277, 18.2%), Logic (43/277, 15.5%), Action-Adventure (35/277, 12.6%) and Strategy (30/277, 10.6%; Table 2).

The top 10 games and game series mentioned by participants were *Mario* (43/473, 9.1%), *Call of Duty* (29/473, 6.1%), *Candy Crush* (28/473, 5.9%), *Fallout* (26/473, 5.5%), *Grand Theft Auto* (26/473, 5.5%), *Final Fantasy* (24/473, 5.1%), *Skyrim* (20/473, 4.2%), *Red Dead* (13/473, 2.7%), *Borderlands* (11/473, 2.3%), and *Sims* (11/473, 2.3%). These games are in the genres of Action (*Mario*, *Call of Duty*, and *Grand Theft Auto*), Role-Play (*Fallout*, *Final Fantasy*, *Skyrim*, *Red Dead*, and *Borderlands*), Simulation (*Sims*), and Logic (*Candy Crush*). Of the top 10 games listed, 1 (10%) was a smartphone-based app game (*Candy Crush*), whereas 9 (90%) were console-based games requiring devices such as a PlayStation, Xbox, or computer to play, although several of the games could be played across multiple platforms.

**Table 1:** Genres of games endorsed by smokers (N=473).^a^

Genre	Participants, n (%)
Action	162 (34.2)
Role-Playing	113 (23.9)
Action-Adventure	101 (21.4)
Logic	79 (16.7)
Strategy	38 (8)
Sports	35 (7.4)
Simulation	28 (5.9)
Casino	24 (5.1)
Board or Card games	17 (3.6)
Massive Multiplayer Online	4 (0.8)
Adventure	3 (0.6)

^a^Due to participants often listing multiple games in different genres, the total in the figure exceeds the total number of participants in the study (N=473).

**Table 2:** Game titles reported by genre (n=277).^a^

Genre	Game titles reported, n (%)
Action	57 (20.6)
Role-Playing	51 (18.4)
Logic game	43 (15.5)
Action-Adventure	35 (12.6)
Strategy	30 (10.8)
Sports	19 (6.9)
Casino games	13 (4.7)
Simulation	13 (4.7)
Board or Card games	10 (3.6)
Adventure	3 (1.1)
Massive Multiplayer Online	3 (1.1)

^a^Logic and Casino games were predominately mobile phone apps such as *Angry Birds* or *Slots*.

### Demographic Variables and Genre Preference

**Figure 1:**
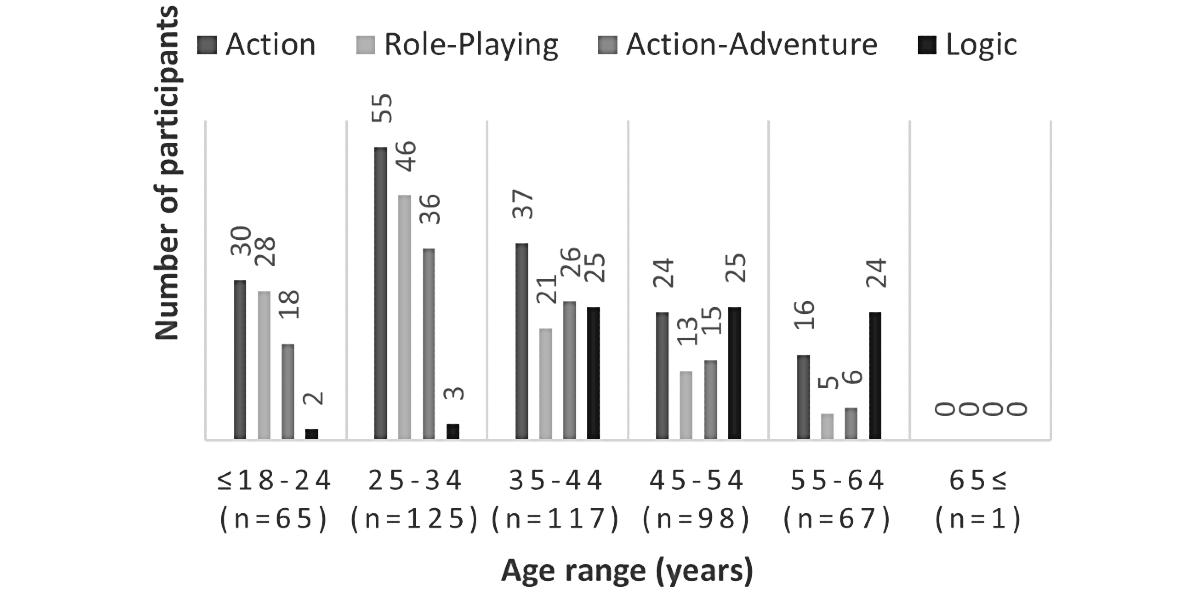
A bar graph depicting the raw number of times a genre was endorsed across age groups. Action, Action-Adventure, Role-Playing, and Logic were selected for this distribution because they were the four most frequently endorsed genres, overall. All 4 genres were significantly correlated with age with *α* (*P*<.01); Action (*P*=.002), Action-Adventure (*P*=.008), Role-Playing (*P*<.001), and Logic (*P*<.001).

Action and Action-Adventure games were the most endorsed across age ranges <18 to 35 years (44%-46%), whereas Logic games were the least endorsed in this age range (2%-3%). Logic games were more frequently endorsed by older participants (aged 35-65 years; 21%-36%). Action games remained endorsed across these older age ranges as well, and it was the second most endorsed category among participants aged 45 to ≥65 years (24%-32%). Figure 1 shows a full distribution of genres endorsed by age. Using Fisher exact test, Action (*P*=.002), Action-Adventure (*P*=.008), Role-Playing (*P*<.001), and Logic (*P*<.001) genres were all significantly related with age. Genres not shown in Figure 1 (Strategy [*P*=.007], Board/Card games [*P*=.001], and Casino games [*P*<.001]) were also significantly related with age. Casino games were endorsed by 8.4% (24/283) of participants aged ≥35 years, and Board/Card games were endorsed by 5.6% (16/283) of the same age range. Strategy games were endorsed more frequently by participants in younger age groups, with 18.5% (7/39) of participants aged ≤18 to 24 years and 10.4% (13/125) of participants aged 25-34 years reporting games in these categories, whereas fewer endorsed strategy games across the 35-44 years (7/117, 5.9%), 45-54 years (5/98, 5.1%), and 55-64 years age ranges (1/67, 1.5%). There were no significant relationships by age for Adventure, Simulation, Sports, and MMO genres. Additional Fisher exact tests were performed to compare smoking intensity with genre preference. Participants were classified by the number of self-reported cigarettes smoked per day as light (<1 to 5 cigarettes per day), moderate (6-10 cigarettes per day), or heavy (>11 cigarettes per day) [26,27]. No significant relationship was found between smoking intensity and genre preference.

## Discussion

### Principal Findings

Our results indicated that smoker video game genre preferences overlap with market information on genre preferences across the population in the United States [22,23]. Furthermore, these data support the existing literature regarding genre preferences that differ across age brackets, consistent with other information about video game preferences in older adults [20,28]. These findings can provide a starting point for researchers seeking to develop smoking cessation games by limiting the scope of *games* broadly defined to a short list of genres and their unique features.

Within each broad genre, there are several unique games with different features that may be more or less amenable to smoking cessation treatment. The Action games that were listed most often featured an avatar that the player used to solve puzzles, defeat enemies, and explore levels through physical challenges and hand-eye coordination [29]. For example, the *Mario* franchise includes a range of games that fall under Action, such as having the player scale platforms in the game world (*Super Mario 64*) to racing (*Mario Kart)* and fighting games (*Super Smash Bros*)*.* Another top game noted by participants was *Call of Duty*, a first-person shooter action game that can be played in a multiplayer and internet-based format, where players shoot at specific targets to achieve an objective. The most common Action games that were listed were primarily single-player games, although some of the games could be played in multiplayer cooperative (ie, players working together with other players to achieve a common goal) and competitive (ie, players working against other players) modes to share the experience with others, either web-based or with a console. Action games use hand-eye coordination, require rapid decision-making, and focus on specific objectives [25]. Ideally, an action game designed to help people quit smoking would be developed so that it is not only a distraction from smoking but also incompatible with smoking (eg, requiring both hands, allowing little opportunity for breaks, and requiring a measure of smoking abstinence to proceed). *Cigbreak* uses a swiping mechanic to break cigarettes, and scores on this task move participants forward on a garden path [7]. The function of this in the game was cue exposure, showing participants images of cigarettes and theoretically reducing the salience of the cue, but may also have been effective because the task was incompatible with smoking. *Inspired* also used a swiping mechanic to sort in-game items rapidly and score points toward revitalizing a lush environment, and later iterations added an action element of defending a tree from invaders [8]. Swiping mechanics combined with the games’ forced landscape mode (ie, holding a phone horizontally with 2 hands) were specifically implemented in *Inspired* to compete with smoking behavior by occupying the participants’ hands with a game instead.

It is possible that Action games might also produce stress, and stress has been shown to serve as a smoking trigger for some individuals [30]; however, preliminary research suggests that the relationship between video games and increased cortisol levels is weak [31-33]. Thus, stress induced by video games may not meet the threshold to trigger cigarette cravings, but further research on the relationship between video games and stress is needed to determine how a high-stress action game might impact smoking. Furthermore, there is no current research evaluating whether video games can impact smoking withdrawal; therefore, video game–based smoking cessation interventions that target incompatible behavior may need to be combined with other evidence-based interventions that target nicotine withdrawal. Future research should evaluate the effects of serious games for smoking cessation on withdrawal and determine whether combining a video game–based intervention with other evidence-based strategies differentially impacts withdrawal and long-term treatment outcomes.

Role-Playing games were the second most popular genre endorsed by participants aged ≤35 years. This genre includes games within the *Fallout*, *Final Fantasy*, *Skyrim*, *Red Dead*, and *Borderlands* franchises. Role-Playing games typically feature the player as a character in the game world who grows and develops through game-based experiences. These games usually feature action elements (such as fighting, shooting, and enemy encounters) and involve the player in the game narrative [29]. For example, in *Fallout 4*, the player assumes control of a customizable character who embarks on a journey that will eventually reunite them with their child, as well as fulfilling other plotlines in a postapocalyptic world. To help with this task, players can equip themselves with various weapons, shop for status-boosting supplies in markets, or build a fortress among a myriad of other in-game choices. The strong narrative used in the Role-Playing genre could be harnessed in a game geared toward smoking cessation by connecting the narrative to smoking abstinence (eg, health of an avatar improves or worsens based on smoking status). One smoking cessation game that falls into the Role-Play genre is *QuitIt*, in which the players guide their character through a craving episode by choosing coping strategies [10]. Role-Playing games could also make access to game-based rewards contingent on abstinence (ie, weapons become unlocked with evidence of smoking abstinence). These game-based rewards hold value to players, as evidenced by the 2020 video game industry’s all-time high revenue of US $60.4 billion, with the majority of that revenue being spent on digital content such as in-game currency, avatar accessories, weapons or tools, and *loot boxes* [34-36].

The Action-Adventure genre often incorporates a combination of features, including action, exploration, puzzles, and narrative, to engage players over the course of the game. For example, in the *Legend of Zelda* series, the player uses a character, Link, to explore the world and uncover story points. During this journey, the player can obtain weapons, armor, and other status-boosting items to customize their play style and ultimately inform their interpretation of how they envision Link. As with the Role-Playing games described previously, Action-Adventure games could harness the narrative and use game-based resources as incentives for meeting abstinence goals or for completing other nongame activities that would support abstinence. The narrative elements of both Role-Playing and Action-Adventure genres may be particularly well suited for promoting long-term engagement in a smoking cessation intervention because it has the potential to keep players engaged over extended periods.

Logic games, such as *Candy Crush*, *Angry Birds*, and *Gardenscapes*, were frequently endorsed by participants. These games feature puzzles with different rules and objectives, such as matching 3 or more of the same picture or object (*Candy Crush)* or determining the force, angle, and special power needed to knock down a structure (*Angry Birds*). These games use simple instructions and mechanics and are sometimes referred to as *casual* games because they are easy to learn and fast to play [37]. *Quittr* uses a logic game in the form of hidden objects in the app and another based on city building (simulation) [11]. *Bejeweled Blitz* is another game that falls within this category of *casual* logic games. One study found that most people played *Bejeweled Blitz* for social reasons, but they also reported that middle-aged adults played it as a form of stress relief, which could substitute cigarettes for people who smoke to manage their stress [38]. Older adults reported playing these games more frequently than other age groups, which is consistent with previous literature on the genre preferences of older adults [20,28]. Using casual logic games might be a successful strategy for distracting people during cravings. Casual gaming bouts last for approximately 6-15 minutes according to market research [39], which aligns closely with the duration of smoking cravings, which last for approximately 6-10 minutes [40]. Nicotine has been reported to increase sustained attention and result in faster response times [40-42], and deprivation from nicotine impairs these functions, leading to reports of worsening attention and working memory [43].

People also report playing casual puzzle games to improve cognitive performance [38]. For example, older adults report feeling as though their visuospatial skills and response times improve after playing casual games, but no explicit measures of attention and working memory were collected beyond self-report. For people who smoke to improve their focus and attention, Action and Logic games could potentially mediate the cognitive impairments experienced during a quit attempt. Further research is needed to determine whether different forms of serious games impact cognition during smoking cessation efforts. The relationship between Action games and increased cognitive performance is also supported. Individuals who play Action and Role-Playing games have been shown to outperform nongamers in attentionally demanding tasks [44], and attentional training through video games can improve performance on tasks [45].

Researchers should consider whether the target audience includes gamers who happen to smoke or nongamers who want to quit smoking using a video game intervention but would not play games otherwise. Most games identified in our survey could only be played on a computer or dedicated console, such as PlayStation or Xbox. Health games targeted for regular gamers might be more successful by devoting resources to console-based games. For example, an intervention designed to target players who chain smoke while playing could be console based and include features that would compete with smoking (eg, frequently requiring the use of both hands), making it incompatible or difficult to do while playing.

It should be noted that developing games can be a costly endeavor; some of the games endorsed by participants reported development budgets of US $40-$50 million (*Call of Duty*: *Modern Warfare 2* [46]). On the lower end of the spectrum, independent, smaller developers can expect to spend anywhere from US $50,000 to US $750,000 [47]. Researchers interested in developing health games targeting gamers would be better served to identify the mechanics these gamers enjoy rather than trying to compete with the graphics and complexity of big-budget games. Alternatively, if researchers are interested in leveraging the appeal of big-budget games for smoking cessation, it may be more useful to treat the game itself as a reward for smoking abstinence goals rather than attempting to recreate the gameplay and presentation. For instance, in the case of an individual who chain smokes while gaming, motion tracking software could be used to identify when the individual begins to smoke and pause the game until the individual confirms that appropriate behaviors have been engaged in (putting out the cigarette, disposing of it, or using a coping strategy such as nicotine gum). *Quittr* takes advantage of this model by offering players resources to help quit smoking and using games to distract participants from cravings [11]. Similarly, *Tobbstop* uses minigames to distract from cravings, and accessing these minigames and other smoking cessation resources in the app rewards the user with progress on beautifying their in-app island [9].

Casual mobile games could leverage the portability of mobile games, allowing for better access when an individual is experiencing a craving and in need of an immediate substitution. For example, an app was recently developed to help individuals who smoke access existing games during cravings, with individuals reporting that playing the games moderately helped them cope with cravings [48]. In addition, mobile games might eventually qualify as prescription digital therapeutics, increasing access to digital interventions through Food and Drug Administration authorization and insurance coverage [49].

A few limitations of the current data set are worth noting. First, future research should collect additional demographic information, such as gender and racial identity, to assess how gaming preferences may differ across demographics, similar to those found with age. Second, participants were asked to report their *favorite* games, which may have limited the range of games reported. In addition, because the question soliciting game titles was open ended, some reports were incomplete or incorrect (for instance, *Smash* was often reported instead of *Super Smash Bros*). Third, the researchers used the publisher-stated genre when available, which may not correspond with the definition of these genres by Adams [14]. Genres can be flexible, and games often use elements of different genres in one game, making them difficult to categorize. Fourth, all participants were recruited through social media or other web-based platforms. This biased sample may not be representative of all video game–playing, treatment-seeking individuals who smoke. Finally, these data were collected from July 2018 to September 2019, so the specific game titles might be different today; however, the game genres would likely remain similar. Future research on this topic would benefit from gathering information on specific features that participants like about the games they play to better aid in the development of gaming interventions for smoking cessation.

### Conclusions

The findings of this study highlight the need for researchers to devote time and resources to understanding their target demographic before developing a game. Although there were some general themes and commonly listed game titles, those interests were shown to change with age. On the basis of these findings, researchers seeking to develop games for smoking cessation should leverage the popularity of Action games broadly speaking and Logic games in populations aged ≥36 years because of the popularity of this genre increasing with age. Participants endorsed games with narrative elements (as seen in the popularity of the Role-Play and Action-Adventure genres), suggesting that a game with an interesting story and character cast might maintain engagement and interest in the game among a large proportion of individuals. Finally, it may be possible to match the function for smoking (eg, attention) with the benefits of certain types of gameplay (eg, cognitive enhancement), which would be expected to improve outcomes.

## Abbreviations

MMO: Massive Multiplayer Online
